# Low-intensity open-field blast exposure effects on neurovascular unit ultrastructure in mice

**DOI:** 10.1186/s40478-023-01636-4

**Published:** 2023-09-06

**Authors:** Chao Li, Shanyan Chen, Heather R. Siedhoff, DeAna Grant, Pei Liu, Ashley Balderrama, Marcus Jackson, Amitai Zuckerman, C. Michael Greenlief, Firas Kobeissy, Kevin W. Wang, Ralph G. DePalma, Ibolja Cernak, Jiankun Cui, Zezong Gu

**Affiliations:** 1 Department of Pathology & Anatomical Sciences, University of Missouri School of Medicine, One Hospital Drive, Medical Science Building, M741, Columbia, MO 65212 USA; 2 Truman VA Hospital Research Service, Columbia, MO 65201 USA; 3 Department of Rehabilitation Medicine, The Third Affiliated Hospital, Sun Yat-sen University, Guangzhou, Guangdong 510630 China; 4 Electron Microscopy Core Facility, University of Missouri, Columbia, MO 65211 USA; 5 Charles W. Gehrke Proteomic Center, University of Missouri, Columbia, MO 65211 USA; 6 Department of Neurobiology, Center for Neurotrauma, Multiomics & Biomarkers (CNMB), Neuroscience Institute, Morehouse School of Medicine, Atlanta, GA 30310-1458 USA; 7 Office of Research and Development, Department of Veterans Affairs, Washington, DC 20420 USA; 8 Department of Surgery, Uniformed Services University of the Health Sciences, Bethesda, MD 20814 USA; 9 Department of Biomedical Sciences, Mercer University School of Medicine, Macon, GA 31207 USA; 10 Atlanta VA Medical and Rehab Center, Decatur, GA 30033 USA

**Keywords:** Open-field blast, Mild traumatic brain injury, Neurovascular unit, Transmission electron microscopy, Ultrastructural abnormalities

## Abstract

Mild traumatic brain injury (mTBI) induced by low-intensity blast (LIB) is a serious health problem affecting military service members and veterans. Our previous reports using a single open-field LIB mouse model showed the absence of gross microscopic damage or necrosis in the brain, while transmission electron microscopy (TEM) identified ultrastructural abnormalities of myelin sheaths, mitochondria, and synapses. The neurovascular unit (NVU), an anatomical and functional system with multiple components, is vital for the regulation of cerebral blood flow and cellular interactions. In this study, we delineated ultrastructural abnormalities affecting the NVU in mice with LIB exposure quantitatively and qualitatively. Luminal constrictive irregularities were identified at 7 days post-injury (DPI) followed by dilation at 30 DPI along with degeneration of pericytes. Quantitative proteomic analysis identified significantly altered vasomotor-related proteins at 24 h post-injury. Endothelial cell, basement membrane and astrocyte end-foot swellings, as well as vacuole formations, occurred in LIB-exposed mice, indicating cellular edema. Structural abnormalities of tight junctions and astrocyte end-foot detachment from basement membranes were also noted. These ultrastructural findings demonstrate that LIB induces multiple-component NVU damage. Prevention of NVU damage may aid in identifying therapeutic targets to mitigate the effects of primary brain blast injury.

**Supplementary Information:**

The online version contains supplementary material available at 10.1186/s40478-023-01636-4.

## Introduction

Blast-induced traumatic brain injury (bTBI) is of particular concern among military personnel.

Explosions cause complex blast injuries, including primary, secondary, tertiary, and quaternary blast injuries [1]. Primary blast injury of the brain as a unique clinical entity due to the direct energy transfer of the shockwaves [2] was recently released by the Centers for Disease Control and Prevention (CDC) with the FY2023 ICD-10-CM code S06.8 A [3] for the related classifications of the disease. Mild TBI (mTBI) caused by low-intensity blast (LIB) exposure of service members during deployment and in non-combat settings such as military occupational training has been increasingly recognized [2, 4]. The Traumatic Brain Injury Center of Excellence (TBICoE), US Defense Department (DoD), recently reported that 82.3% of 453,919 military TBIs during the interval 2000–2021 Q4 were mTBI [5]. This injury, most often without loss of consciousness, associates with a greater than a two-fold increase in the risk of later dementia [6]. Lifelong disabilities impose immense burden on affected Service members and Veterans, their families, and society. An urgent need to better understand and treat the consequences of these “invisible injuries”.

The neurovascular unit (NVU) is an anatomical and functional structure comprised of multiple components, including endothelial cells, tight junctions, pericytes, basement membranes, perivascular astrocytes, microglia, neurons, and extracellular matrix. The NVU is central to the interactions between microvessels and neuronal cells in regulating cerebral blood flow and maintaining the integrity of the blood–brain barrier (BBB) [7, 8]. Disturbances of NVU homeostasis occur in many neurological diseases, including ischemic stroke and TBIs [9–14]. Increased BBB permeability induced by single or repeated blast exposure (shock tubes at 72 to 137 kPa) has been documented by leakage of exogenous chemical tracers, proteomic analyses, and MRI imaging [15–19]. Structural abnormities of the NVU occurred post-blast exposure generated in shock tubes (74.5 kPa), including swollen astrocyte end-feet, disrupted pericytes and endothelial cells, as well as abnormally shaped capillary lumens [20, 21]. Blast-induced NVU impairments have been postulated to include oxidative stress, matrix metalloproteinases (MMP) activation, inflammation, and increased glial cell reactivity [19, 20, 22].

Our previous observation using the open-field LIB mouse model—entailing elimination of head movement—in an open-field environment revealed that single blast exposure with a peak overpressure of 46.6 kPa did not result in gross microscopic damage or necrosis in the brain, while TEM identified multiple ultrastructural abnormalities of axonal myelin sheaths, mitochondria and synapses [23–25]. This study provides quantitative and qualitative TEM analyses of multi-component NVU abnormalities uniquely induced by open-field LIB exposure. We detailed morphologic features and quantitative analyses of the microvessel lumens, pericytes, endothelial cells, basement membranes, astrocyte end-feet, and tight junctions using TEM at 7- and 30-days DPI, as well as proteomic assays at 24 h post-injury. We also quantified neurofilaments light chain (Nf-L) and glial fibrillary acidic protein (GFAP) in mouse brain tissues and plasmas at acute, subacute and chronic stages post-LIB injury, which were also used in testing patients with TBI as the FDA-approved peripheral blood biomarkers [26, 27]. In combining these multidisciplinary assessments, we hypothesized that a single LIB in open field results in ultrastructural NVU impairments.

## Materials and methods

### Animals and open-field LIB setting

All animal experiments were performed in accordance with the University of Missouri-approved protocols for the Care and Use of Laboratory Animals and the Animal Research: Reporting of In Vivo Experiments (ARRIVE) guidelines. Two-month-old male C57BL/6J mice (The Jackson Laboratory, Bar Harbor, ME) were used. Mice were housed in groups of 5 per cage with a 12-hour light / dark cycle in home-cages containing bedding, with *ad libitum* access to food and water. LIB exposure was conducted at the Missouri University of Science & Technology, as previously reported [23–25, 28–32]. Animals were randomly assigned into the LIB-exposed group and the sham group. The investigators performing experimental analyses were double-blinded about the group assignments of the mice. Mice were anesthetized with an intraperitoneal (i.p.) injection of 5 µL / g body weight of ketamine / xylazine mixture (25 mg / mL ketamine and 1.25 mg / mL xylazine). Mice were placed in metal mesh animal holders in prone orientation at 3-meter distance away from the detonation of a 350 g C4 explosive. The explosion generated a magnitude of 46.6 kPa peak overpressure with a maximal impulse of 60 kPa × ms. No mouse head- or body-movements were observed. Thus, the resulting LIB-induced TBI was, in all likelihood, attributable to the primary blast effects, but not inertial brain injury. The sham group underwent identical handling and anesthesia procedures, only without LIB exposure. After recovery from anesthesia, mice were monitored for at least 15–30 min and allowed access to food and water *ad libitum*.

### TEM imaging

Animals used for the TEM study were sacrificed at 7 and 30 DPI. Samples were collected and processed as previously described [24, 25]. Briefly, anesthetized mice were perfused transcardially with saline containing heparin at room temperature, and then with cold 4% paraformaldehyde. One-mm thick brain sections were cut with the brain matrix (Ted Pella, Inc., Altadena, CA, USA) and fixed with primary fixative solution (100 mM sodium cacodylate, 2% glutaraldehyde, and 2% paraformaldehyde, Electron Microscopy Sciences, Hartfield, PA, USA). Brain sections were further stereologically cut into 1 mm^3^ tissue from cerebral cortices under the light microscope. Tissues were stored in 0.1 M NaCacodylate buffer (pH = 7.4) containing 0.13 M sucrose and then embedded with 1% osmium tetroxide (Ted Pella, Inc. Redding, California) in Cacodylate buffer. Each tissue block was trimmed approximately 150 μm deep and the surrounding tissues to avoid potential artifacts. Approximately 250 μm × 200 μm tissue blocks were selected for sectioning at 85 nm thickness using an ultramicrotome (Ultracut UCT, Leica Microsystems, Germany) and a diamond knife (Diatome, Hatfield PA). Images were acquired with a JEOL JEM 1400 transmission electron microscope (JEOL, Peabody, MA) at 80 kV on a Gatan Ultrascan 1000 CCD (Gatan, Inc, Pleasanton, CA).

### Quantitative analyses of NVU components in TEM images

Electron micrographs of microvessels from mouse brains were collected for analysis using Image J software. Criteria for microvessel selections and quantitative methods were based on previous reports [33–37]. Inclusion criteria for microvessels included: (1) intact vessels; (2) diameters < 8 mm; and (3) images not containing nuclei of endothelial cells or pericytes. Numbers of microvessels meeting criteria and included for quantitative analyses: 7-day sham, n = 27 from 2 mice; 7-day LIB, n = 48 from 4 mice; 30-day sham, n = 32 from 3 mice; 30-day LIB, n = 33 from 4 mice. Microvessel lumens, endothelial cells, pericytes, and astrocyte end-feet were manually traced using Image J software to calculate areas. Relative areas of endothelial cells, pericytes, and astrocyte end-feet were normalized with lumen areas of individual microvessels, with the formulas as follows:

*Relative pericyte areas = pericytes areas / lumen areas;*

*Relative endothelial cell areas = endothelial cell areas / lumen areas;*

*Relative astrocyte end-foot areas = astrocyte end-foot areas / lumen areas.*

Lumen ratios were defined as the longest diameter in the lumen circle / the perpendicular bisector of the long diameter. Pericyte coverages of endothelial cells were calculated as the percentages of total lengths of inner pericyte processes around each microvessel relative to perimeters of endothelial cells. Astrocyte end-foot coverages of the microvessels were calculated as the percentages of the total length of inner end-foot processes around each microvessel relative to the perimeters of the basement membranes. Vacuolations of the endothelial cells were quantified by the numbers of vacuoles (diameters > 50 nm) per 10 μm of the endothelial cell circumferences. Basement membrane thickness was measured in straight lines between the inside and outside edges of the basement membranes at four cardinal points. Cardinal points were chosen typically at the 3, 6, 9, and 12 o’clock positions, avoiding areas where the basement membranes have been expanded by the presence of pericytes. Tight junctions were visualized in high-magnification images: 7-day sham, n = 25 from 2 mice; 7-day LIB, n = 55 from 4 mice; 30-day sham, n = 41 from 3 mice; 30-day LIB, n = 33 from 4 mice. Tight junctions with discontinuities, membrane-bound spaces, and vacuole formations were classified as abnormal.

### Immunofluorescence

Animals used for the immunofluorescence study were sacrificed at 7 DPI (sham, n = 6; LIB, n = 6). Mouse brains were dissected and cut into serial coronal sections of 40-µm thickness, then post fixed with 4% paraformaldehyde as previously described [11, 13]. Sections were immunostained with the following antibodies: aquaporin-4 (AQP-4) (A5971, MilliporeSigma, Burlington, MA, USA), glial fibrillary acidic protein (GFAP) (G3893, MilliporeSigma, Burlington, MA, USA), goat anti-rabbit Alexa488 (A-110,034, Thermo Fisher Scientific, Waltham, MA, USA) and goat anti-mouse Alexa594 (A-11,005, Thermo Fisher Scientific, Waltham, MA, USA), as well as nuclear DNA dye Hoechst 33,342. Fluorescence images were taken with a Leica DMI 6000B microscope (Leica Microsystem). 3D deconvolution was used to enhance the sharpness and contrast of fluorescent images with the LAS AF analysis tools.

### Protein extraction, digestion, and mass spectrometry (MS) analysis

Animals used for the proteomic study were sacrificed at 24 h post-injury (sham, n = 5; LIB, n = 5). Mouse brains were dissected and processed as described previously [29]. The temporal halves of the cortex tissues were collected for analysis. Lyse sample buffer (2% sodium dodecyl sulfate [SDS], 0.5 M tetraethylammonium bicarbonate [TEAB], pH 8.5, protease inhibitor cocktails) was added to each tissue sample. Samples were homogenized using Glas-Col stringer 099 C K43 (Glas-Col LLC, IN) and then centrifuged at 17,000 × g for 20 min at 4 °C. Supernatants were collected and then precipitated by cold acetone. Protein pellets were suspended in 6 M urea, 2 M thiourea, and 100 mM ammonium bicarbonate. Protein concentrations were determined using Pierce 660 nm Protein Assay (Thermo Fisher Scientific) according to the manufacturer’s protocol. An equal amount of protein (30 µg) from each sample was reduced, alkylated, digested by sequential LysC, and trypsin, and purified by C18 Tips as described previously [38]. Purified peptides were then lyophilized and resuspended in 5% acetonitrile, 0.1% formic acid. Resuspended peptides were analyzed using Bruker timsTOF pro mass spectrometer as previously described with 90 min LC gradient: initial condition 2% B (A: 0.1% formic acid in water, B: 99.9% acetonitrile, 0.1% formic acid), followed by 26 min ramp to 17% B, 17–25% B over 36 min, 25–37% B over 15 min, a gradient of 37% B to 80% B over 6 min, hold at 80% B for 7 min [38]. The timsTOF pro was operated in parallel accumulation–serial fragmentation (PASEF) mode. The duty cycle was locked to 100%. Ion mobility coefficient (1 / K_0_) value was set from 0.6 to 1.6 Vs cm^− 2^. MS data were collected over m / z range of 100 to 1700. During MS / MS data collection, each TIMS cycle contained one MS and ten PASEF MS / MS scans. The exclusion was active after 0.4 min.

### Quantification of Nf-L and GFAP levels in brain tissue and plasma

Other cohorts of mice were sacrificed at 24 h, 7 days, and 3 months post-LIB injury. After the collection of circulating blood to prepare plasma, mouse brains were dissected and processed as described previously [29]. The temporal halves of the cortex tissues were collected for biomarker analysis. Brain tissue lysate was prepared by first homogenizing the cortex tissue (about 150–200 mg) with cold lysis buffer (20 mM Tris-HCl, 150 mM NaCl, 1% Triton-X100 (v/v), 5 mM EDTA and 1x protease/phosphatase inhibitor cocktail (Sigma) for 30 s x 2, then centrifuged at 4˚C at 1,500 rpm for 5 min. The supernatant was collected and assayed for protein concentration. Most of the tissue or plasma sample numbers were 4–6 per group and some up to 10 with the detailed sample numbers of each group listed in figure legends. Thereafter the protein concentration was adjusted to 1 µg/µL. For biomarker assay, 25 µL of either tissue lysate or plasm samples were diluted ¼ with sample diluent (Quanterix). We assayed GFAP and Nf-L (Quanterix Simoa) with an ultrasensitive immunoassay using digital array technology (Single Molecule Arrays, SiMoA)-based Human Neurology 4-Plex B assay (N4PB; Item 103,345). SiMoA assays were run on the SR-X benchtop assay platform (Quanterix Corp., Lexington, MA) at the Morehouse School of Medicine laboratory (Atlanta, GA) accordingly to the manufacturer’s instructions [26, 27]. The lower limit of quantitation (LLOQ), limit of detection (LOD), and dynamic range are 9.38 pg/mL, 1.32 pg/mL, and 1.32 to 250,000 pg/mL for GFAP. Interassay and intraassay % Coefficients of Variability (CV) are 7.5–10.8% for GFAP. The LLOQ, LOD, and dynamic range are 0.625 pg/mL, 0.097 pg/mL, and 0.0971-10,000 pg/mL for Nf-L, respectively. Interassay and intraassay % CV are 4.6–6.9% and 3.5–7.5%, respectively for Nf-L.

### Statistical analyses

Data were analyzed using Prism software 9 (GraphPad Software, La Jolla, CA). Sample size determination was based on previous publications [24, 25]. For statistical analyses of TEM studies, the D’Agostino-Pearson test was used for normality as recommended by Prism. Data adhering to normal distribution were analyzed with unpaired two-tailed *t*-test and depicted as mean ± SD; data adhering non-normally distributed data were analyzed with two-tailed Mann-Whitney nonparametric tests and presented as the median and interquartile range (being the 25th and 75th percentile). For statistical analyses of proteomic datasets, the Shapiro-Wilk test was used for normality as the appropriate method for small sample sizes. Data adhering to normal distribution were analyzed with unpaired two-tailed *t*-test and depicted as mean ± SD. For statistical analyses of Nf-L and GFAP levels, the Shapiro-Wilk test was used for normality as the appropriate method for small sample sizes. Data adhering to normal distribution were analyzed with unpaired two-tailed *t*-test and depicted as mean ± SD; data adhering to non-normally distributed data were analyzed with two-tailed Mann-Whitney nonparametric tests and presented as the median and interquartile range (being the 25th and 75th percentile). Differences were considered significant at p < 0.05.

## Results

### Luminal irregularities of microvessels in mouse brains post-LIB exposure

**Fig. 1:**
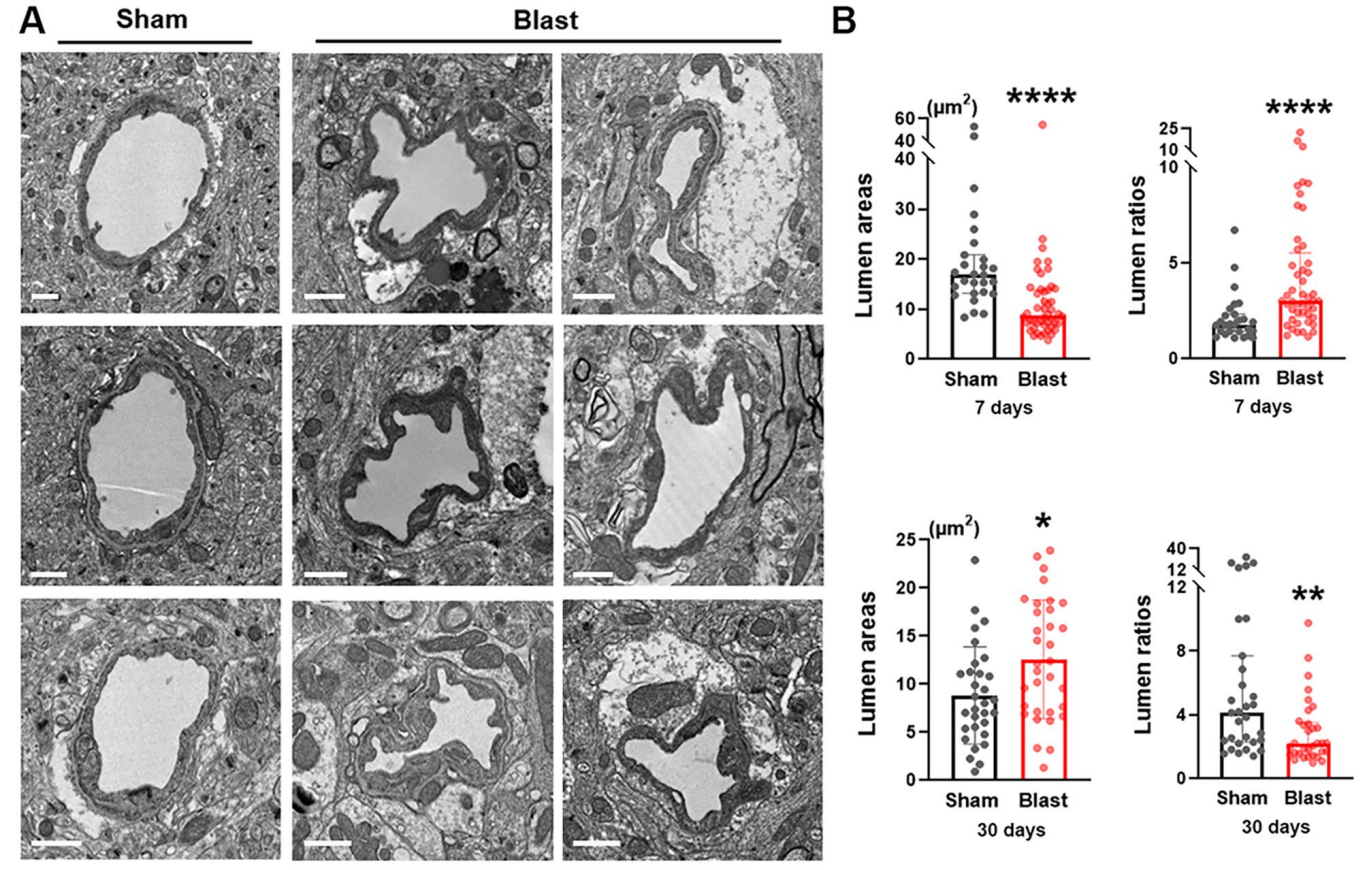
Ultrastructural abnormalities of luminal circularities in mouse brains post-LIB exposure. (**a**) Representative electron micrographs of microvessels in sham controls and LIB-exposed mice at 7 DPI. Scale bar, 1 μm. (**b**) Quantifications of electron micrographs reveal lumen areas and lumen ratios in sham controls and LIB-exposed mice at 7 and 30 DPI. *, p < 0.05, **, p < 0.01 and ****, p < 0.0001. Lumen areas at 30 DPI are expressed as mean ± SD; other data are expressed as median and interquartile range

Abnormal constrictions of blood vessels (vasospasms) in brains have been well documented in ischemic stroke, as well as TBIs with incidences ranging from 19 to 68% [39, 40]. The present ultrastructural study showed that microvessels in LIB-exposed mouse brains exhibited varying irregularities of luminal circularity compared to sham controls at 7 DPI (Fig. 1a). Detailed quantitative analyses revealed that microvessel lumen areas were significantly smaller by 42.0% (mean value) and lumen ratios significantly higher by 116.0% (mean value) in LIB-exposed mice compared to sham controls at 7 DPI. Interestingly, at 30 DPI these patterns were not identical, however significantly larger lumen areas and lower lumen ratios in LIB-exposed mice were observed (Fig. 1b).

In a continuum of biomarkers released at different time points following the TBI, BBB damage-related proteins can be identified in the acute phase (within 24 h) post injury [41].

To further investigate molecular biosignatures of instant reactions of LIB exposure leading to the luminal irregularities, profiles of global proteomes were analyzed using label-free quantitative mass spectrometry in brain samples collected at 24 h post-LIB exposure. Identified proteins were examined by searching the published proteome of mouse cerebral arteries [42]. Four proteins involved in vasomotor functioning were significantly downregulated in LIB-exposed mice: nitric oxide synthase (NOS) (S4R255), tyrosine 3-monooxygenase (TY3H), serine / threonine-protein phosphatase (fragment) (A0A0G2JFF1) and serine / threonine-protein phosphatase PP1-alpha catalytic subunit (PP1A). In addition, two proteins involved in vasomotor were significantly upregulated in LIB-exposed mice, including calcium-transporting ATPase, two isoforms (F8WHB1 and S4R1C4) (Supplemental Fig. 1).

### Degeneration of pericytes in mouse brains post-LIB exposure

**Fig. 2:**
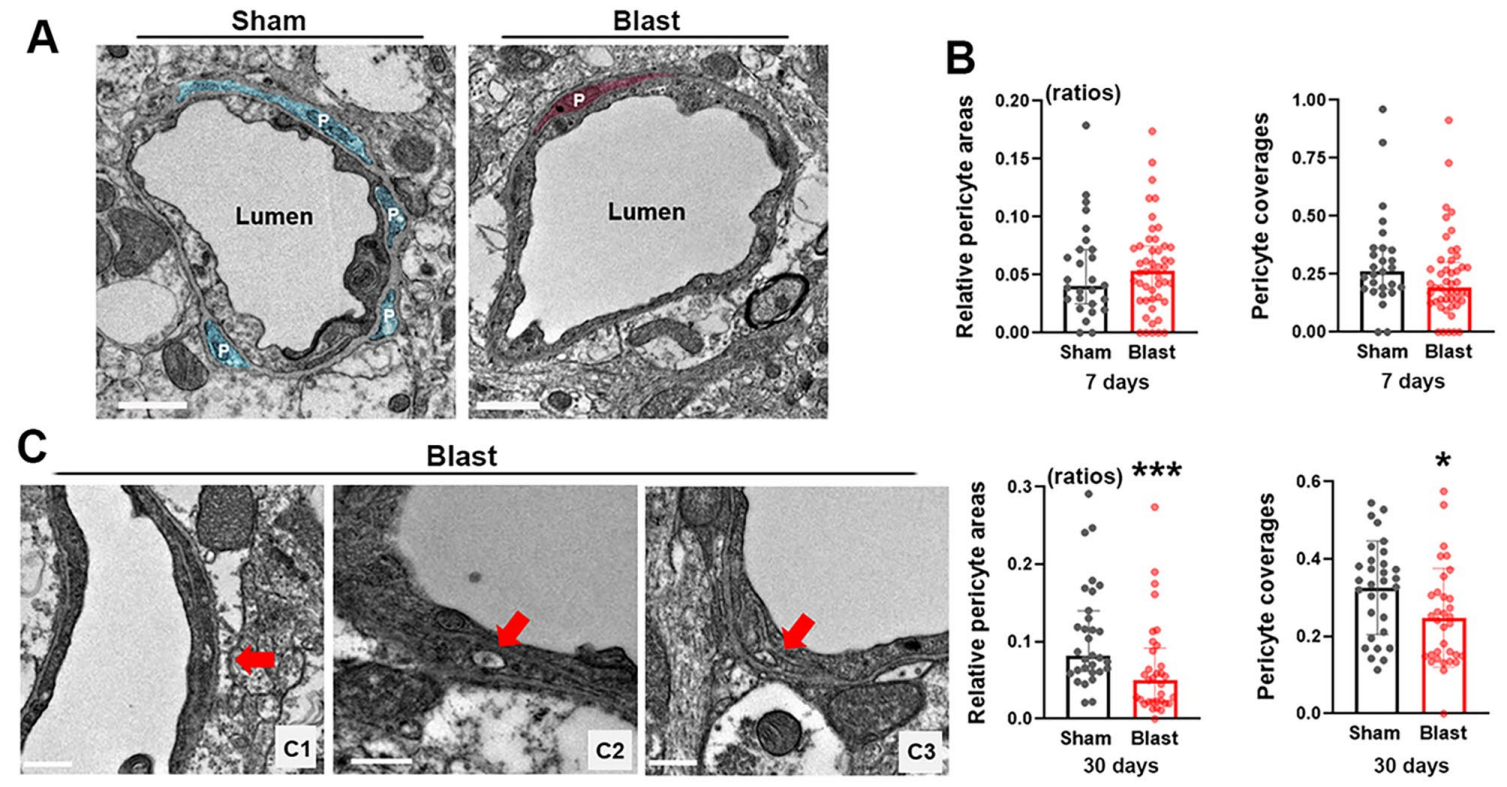
Ultrastructural abnormalities of pericytes in mouse brains post-LIB exposure. (**a**) Representative electron micrographs of microvessels in sham controls and LIB-exposed mice. Pericytes (P) are highlighted in blue in sham controls and pink in LIB-exposed mice at 30 DPI. Scale bar, 1 μm. (**b**) Quantifications of electron micrographs reveal pericyte coverages and relative pericyte areas in sham controls and LIB-exposed mice at 7 and 30 DPI. *, p < 0.05 and ***, p < 0.001. Pericyte coverages at 30 DPI are expressed as mean ± SD; other data are expressed as median and interquartile range. (**c**) Red arrows indicate vacuoles within pericytes in LIB-exposed mice at 7 (c_1_) and 30 (c_2_, c_3_) DPI. Scale bar, 0.5 μm

Pericytes regulate diameters and blood flow in brain microvessels [43, 44]. Literature reports that pericytes cover approximately 30% of the outer perimeters of endothelial cells in adult mice [45], which is consistent with the 31.5% average pericyte coverage in sham control mice of our current study (Fig. 2b). Loss of pericytes is involved in the pathophysiological processes of neurodegenerative diseases, such as poststroke dementia, vascular dementia, and Alzheimer’s disease [46]. The present ultrastructural study showed pericytes in LIB-exposed mice did not differ significantly from sham controls at 7 DPI (Fig. 2b). By contrast, relative pericyte areas were significantly less by 40.2% (mean value) and pericyte coverages significantly reduced by 23.7% (mean value) in LIB-exposed mice compared to sham controls at 30 DPI (Fig. 2a and b). Vacuole formations were also observed in pericytes in LIB-exposed mice at 7 and 30 DPI (Fig. 2c_1 − 3_).

### Endothelial cell swellings in mouse brains post-LIB exposure

**Fig. 3:**
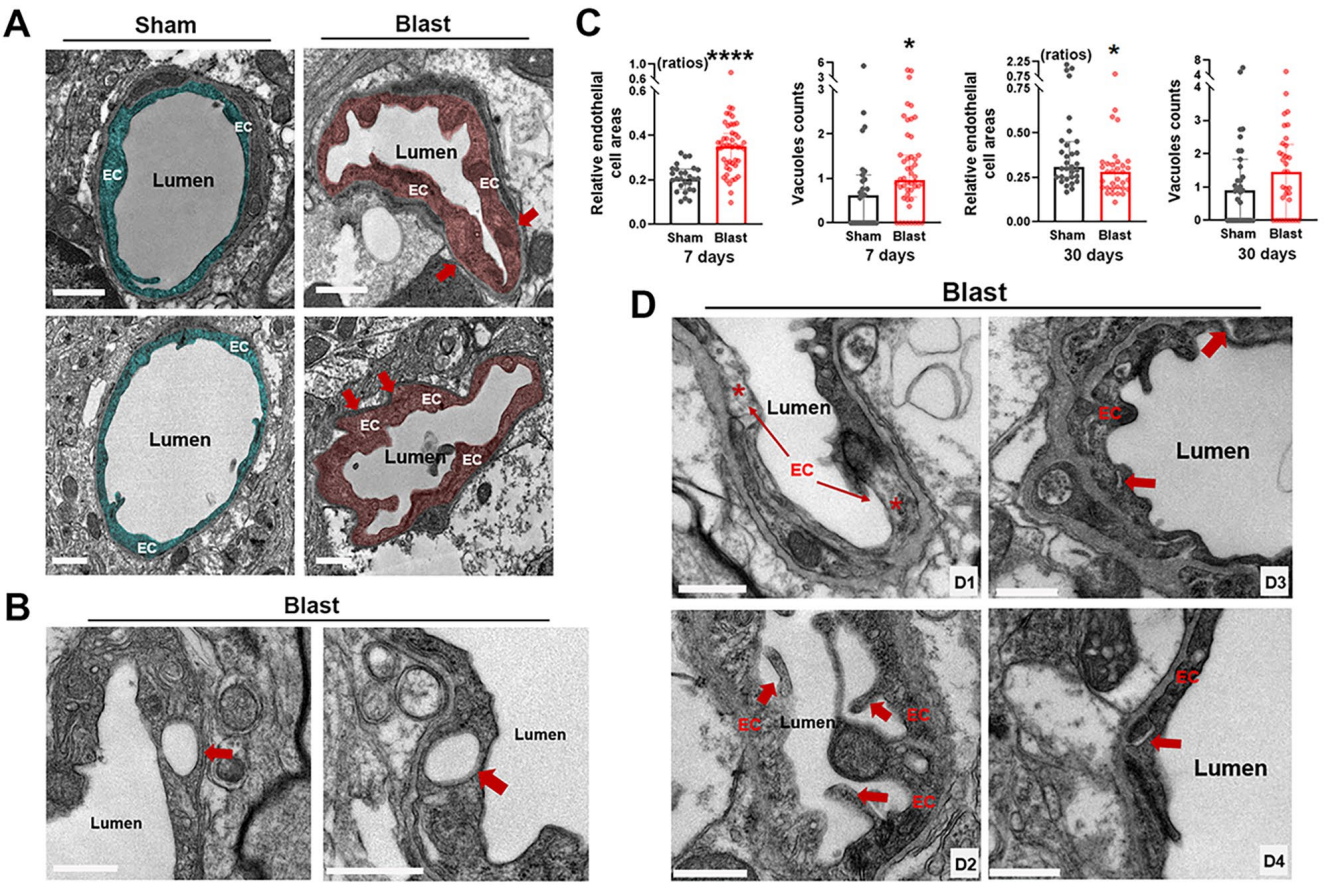
Ultrastructural abnormalities of endothelial cells in mouse brains post-LIB exposure. (**a**) Representative electron micrographs of microvessels in sham controls and LIB-exposed mice. Endothelial cells (EC) are highlighted in blue in sham controls and pink in LIB-exposed mice at 7 DPI. Scale bar, 1 μm. (**b**) Red arrows indicate vacuoles within endothelial cells in LIB-exposed mice at 7 DPI. Scale bar, 1 μm. (**c**) Quantifications of electron micrographs reveal relative endothelial cell areas and densities of vacuoles in sham controls and LIB-exposed mice at 7 and 30 DPI. *, p < 0.05 and ****, p < 0.0001. Data are expressed as median and interquartile range. (**d**) Red arrows and red asterisks indicate ultrastructural abnormalities of endothelial cells (EC) in LIB-exposed mice, including density inconsistencies (d_1_) and abnormal bulges (d_2_) at 7 DPI, as well as endothelial cell detachments (d_3_ at 30 DPI and d_4_ at 7 DPI). Scale bar, 0.5 μm

Endothelial cells are a single layer of flat cells covering the inner surface of blood vessels. Swellings of endothelial cells have been identified in cerebral ischemia and associated with the breakdown of the BBB [47]. The present ultrastructural study showed endothelial cell swellings and vacuole formations in LIB-exposed mice at 7 DPI (Fig. 3a and b). Quantitative analyses revealed significantly larger relative endothelial cell areas by 67.2% (mean value) and significantly higher densities of vacuoles by 55.4% (mean value) in LIB-exposed mice compared to sham controls at 7 DPI (Fig. 3c). By contrast, LIB-exposed mice showed slightly reduced relative endothelial cell areas and no significant differences in vacuole densities at 30 DPI (Fig. 3c). We also identified other ultrastructural abnormalities of endothelial cells in LIB-exposed mice, including inconsistent densities of endothelial cells, abnormal internal bulges, and detachments of endothelial cells at 7 and 30 DPI (Fig. 3d_1 − 4_).

### Thickening of basement membranes in mouse brains post-LIB exposure

**Fig. 4:**
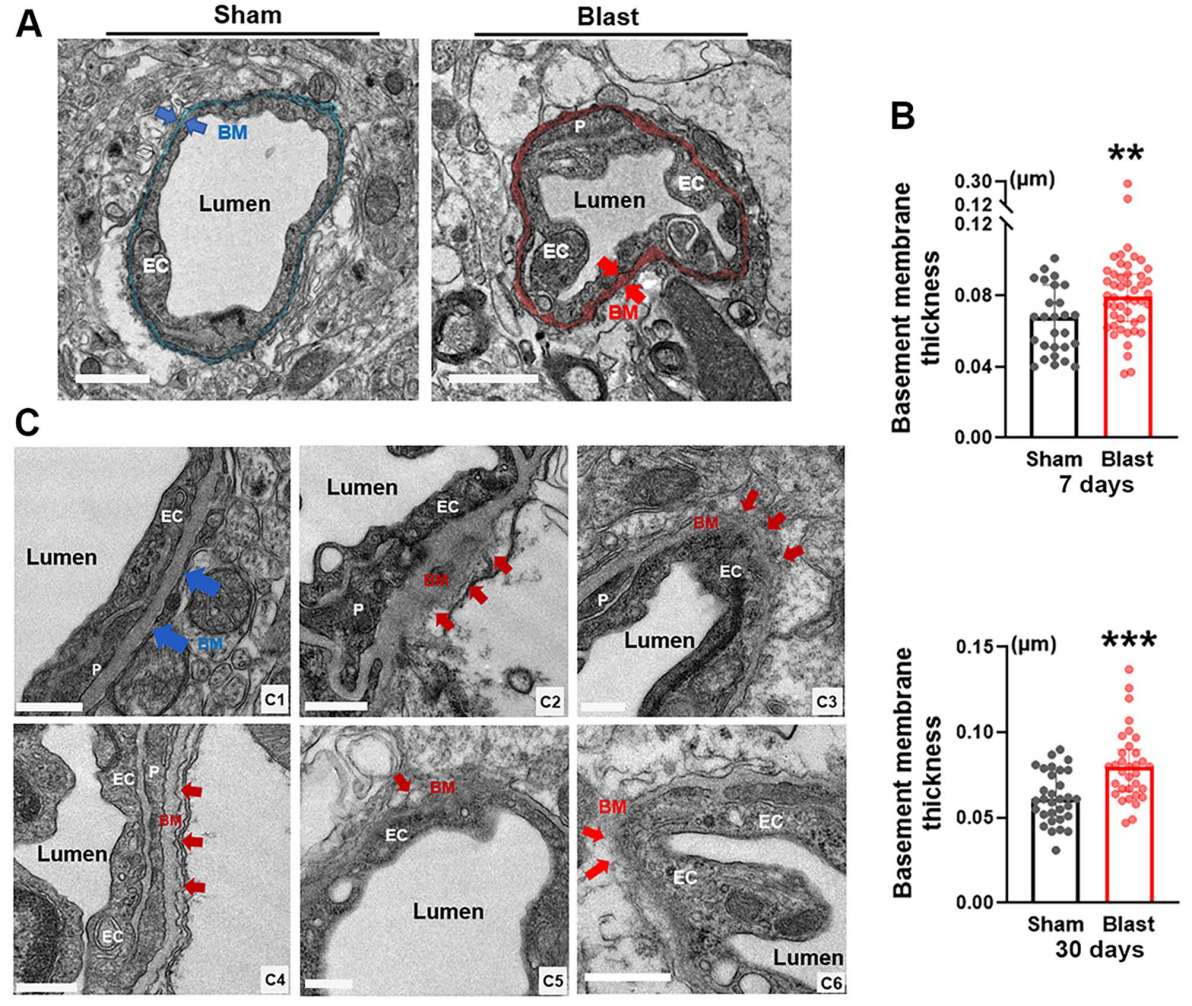
Ultrastructural abnormalities of basement membranes in mouse brains post-LIB exposure. (**a**) Representative electron micrographs of microvessels in sham controls and LIB-exposed mice. Basement membranes (BM) are highlighted in blue in sham controls and pink in LIB-exposed mice at 7 DPI. Scale bar, 1 μm. (**b**) Quantifications of electron micrographs reveal basement membrane thickness in sham controls and LIB-exposed mice at 7 and 30 DPI. **, p < 0.01 and ***, p < 0.001. Data are expressed as median and interquartile range. (**c**) Blue arrows indicate normal basement membrane (BM) structure in sham control mice (c_1_); red arrows indicate abnormal basement membrane structures in LIB-exposed mice with bulges (c_2_), fragmentations (c_3_), corrugations (c_4_), and vacuoles within basement membranes (c_5_) at 7 DPI, as well as fragmentations (c_6_) at 30 DPI. Scale bar, 0.5 μm

Structural changes of the basement membranes of NVU, including degradation, diffusion, electron-lightening, and thickening have been identified as important features seen in neurodegenerative diseases, such as ischemic stroke [48]. In this study assessing ultrastructural changes, LIB exposure led to thickening of basement membranes (Fig. 4a). Significantly thicker basement membranes of 0.084 and 0.080 μm (mean value) were found in LIB-exposed mice at 7 and 30 DPI, compared to 0.065 and 0.062 μm (mean value) in sham control mice respectively (Fig. 4b). In addition, compared to basement membranes in sham control mice (Fig. 4c_1_), multiple ultrastructural abnormalities were also observed in LIB-exposed mice, including bulges, fragmentations, corrugations, and vacuole formations at 7 and 30 DPI (Fig. 4c_2 − 6_).

### Astrocyte end-foot swellings and detachments in mouse brains post-LIB exposure

**Fig. 5:**
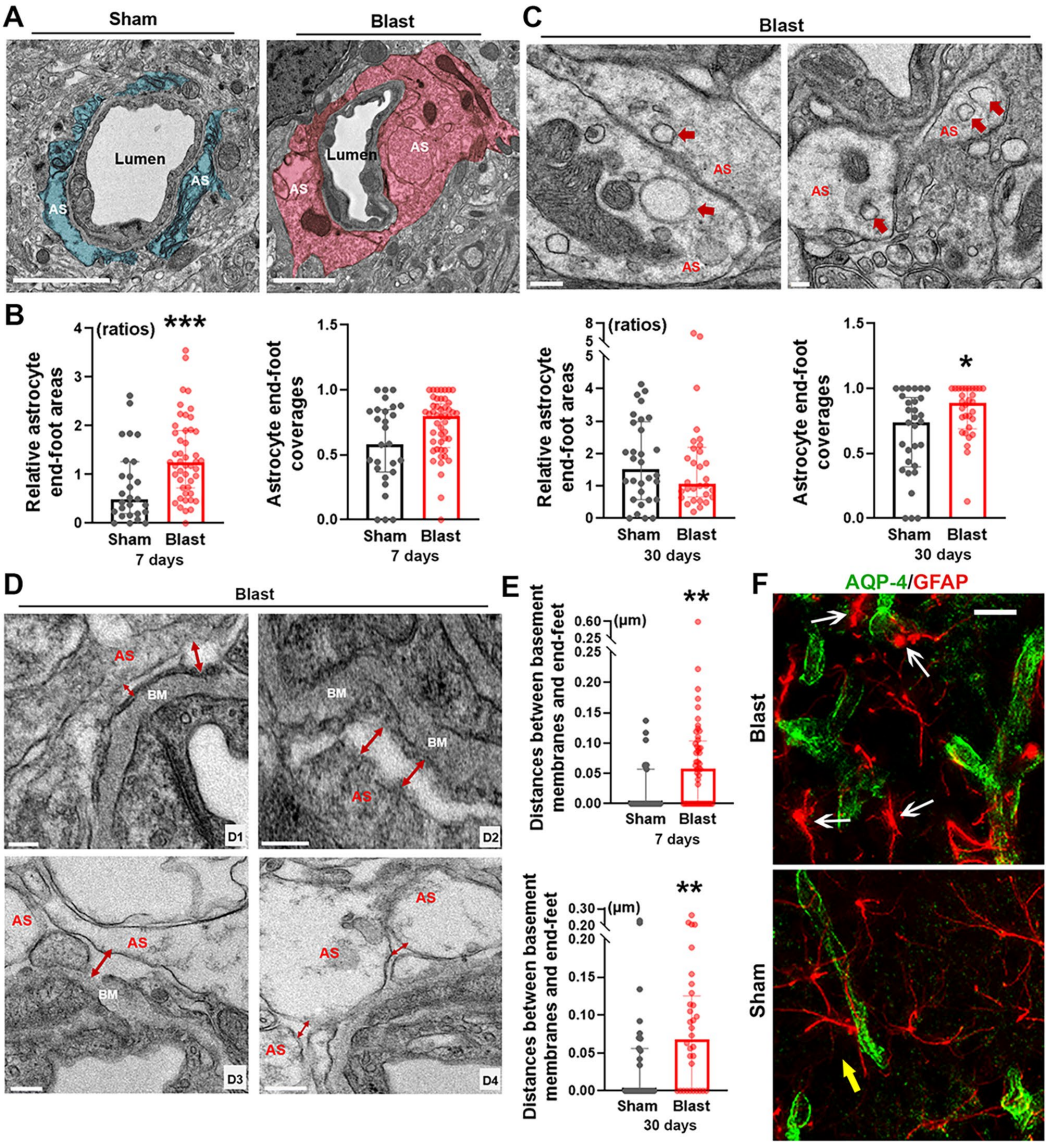
Ultrastructural abnormalities of astrocyte end-feet and perivascular astrocyte reactivity in mouse brains post-LIB exposure. (**a**) Representative electron micrographs of microvessels in sham controls and LIB-exposed mice at 7 DPI. Astrocyte end-feet (AS) are highlighted in blue in sham controls and pink in LIB-exposed mice. Scale bar, 2 μm. (**b**) Quantifications of electron micrographs reveal astrocyte end-foot coverages and relative astrocyte end-foot areas in sham controls and LIB-exposed mice at 7 and 30 DPI. *, p < 0.05 and ***, p < 0.001. Data are expressed as median and interquartile range. (**c**) Red arrows indicate vacuoles within astrocyte end-feet (AS) in LIB-exposed mice at 7 DPI. Scale bar, 0.2 μm. (**d**) Red arrows indicate enlarged spaces between basement membranes (BM) and astrocyte end-feet (AS) (d_1 − 3_) and between adjacent end-feet (d_4_) in LIB-exposed mice at 7 DPI. Scale bar, 0.1 μm d_1_, d_2_ and 0.2 μm d_3_, d_4_. (**e**) Quantifications of electron micrographs reveal distances between basement membranes and astrocyte end-feet in sham controls and LIB-exposed mice at 7 and 30 DPI. **, p < 0.01. Data are expressed as median and interquartile range. (**f**) Photomicrographs of immunofluorescence staining with AQP-4 and GFAP showing perivascular astrocytes at 7 DPI. White arrows indicate reactive astrocytes in LIB-exposed mice, yellow arrows indicate non-reactive astrocytes in sham controls. Scale bar, 15 μm

In the present ultrastructural study, swellings of astrocyte end-feet were observed in LIB-exposed mice at 7 DPI (Fig. 5a), in agreement with previous studies on bTBI and ischemic stroke [21, 49]. Quantitative analyses revealed significantly larger relative astrocyte end-foot areas by 75.9% (mean value) at 7 DPI and significantly increased astrocyte end-foot coverages by 27.4% (mean value) at 30 DPI in LIB-exposed mice compared to sham controls (Fig. 5b). Astrocyte end-feet were also laden with vacuoles in LIB-exposed mice at 7 DPI (Fig. 5c).

We further investigated whether astrocyte end-foot detachments [50–52] were involved in LIB-induced mTBI. We observed enlarged spaces between astrocyte end-feet and basement membranes (Fig. 5d_1 − 3_), as well as between adjacent end-feet (Fig. 5d_4_). Quantitative analyses revealed significantly increased distances between astrocyte end-feet and basement membranes by 218.0% and 133.5% (mean values) in LIB-exposed mice compared to sham controls at 7 and 30 DPI respectively (Fig. 5e).

Reactive astrocytes in response to TBIs occur in neuroinflammation and progression of neurodegenerative diseases, as identified in our previous non-blast TBI study, as well as bTBI studies from other groups [17, 53, 54]. Aquaporin-4 (AQP-4) are water channels located within the astrocyte end-feet surrounding cerebral blood vessels. We studied perivascular astrocytes using the colocalization of AQP-4 and glial fibrillary acidic protein (GFAP) immunoreactivity. In LIB-exposed mice at 7 DPI, perivascular astrocytes showed morphological changes of soma hypertrophy and increased expression of GFAP, while non-reactive astrocytes occurred in sham controls (Fig. 5f).

### Tight junction structural abnormalities in mouse brains post-LIB exposure

**Fig. 6:**
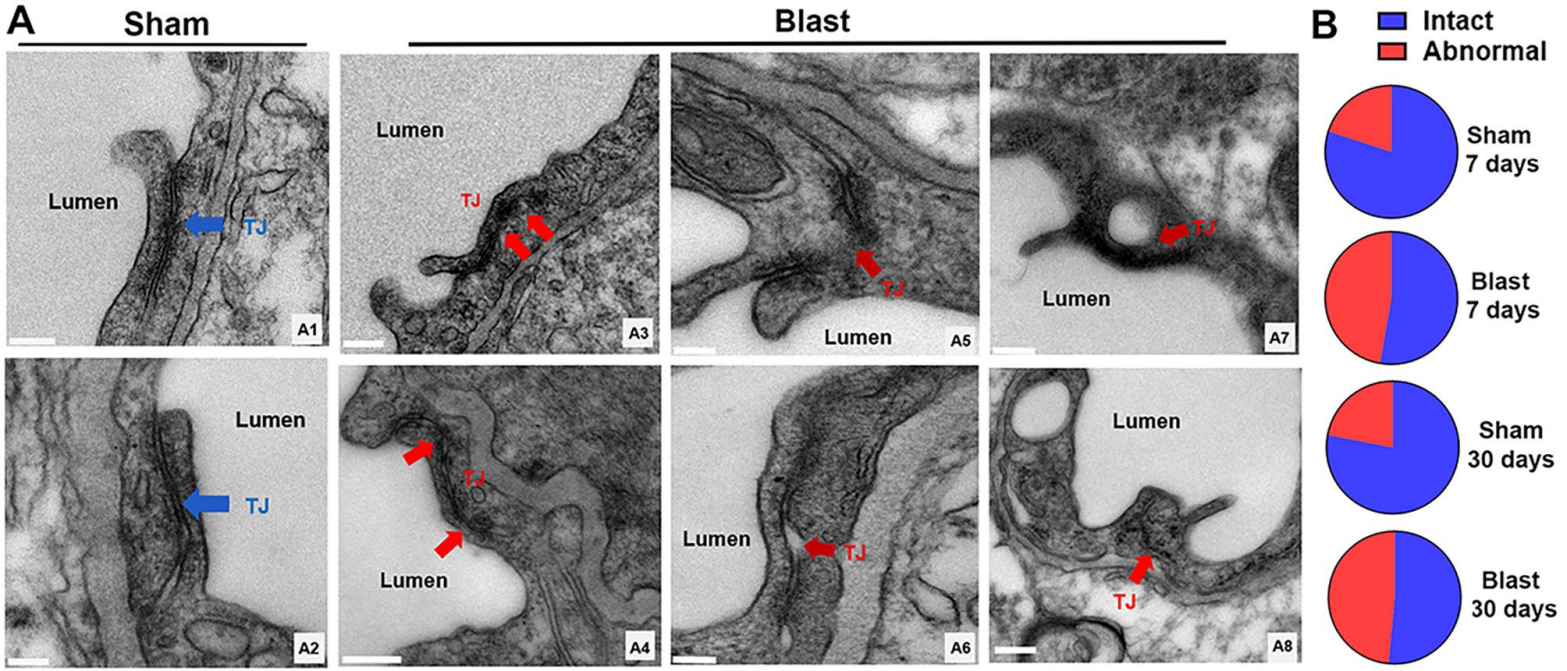
Ultrastructural abnormalities of tight junctions in mouse brains post-LIB exposure. (**a**) Representative high resolution electron micrographs of microvessels in sham controls (a_1_, a_2_) and LIB-exposed mice at 7 (a_4_, a_5_, a_6,_ a_8_) and 30 (a_3_, a_7_) DPI. Tight junctions (TJ) are indicated with blue arrows in sham controls and pink arrows in LIB-exposed mice. Scale bar, 0.2 μm a_1_, a_3_, a_4_, a_5_, a_7_, a_8_ and 0.5 μm a_2_ and a_6_. (**b**) Histograms show the percentages of intact and abnormal tight junctions in sham controls and LIB-exposed mice at 7 and 30 DPI.

Tight junctions connecting adjacent endothelial cells play key roles in maintaining the structural and functional integrity of the BBB [55]. Ultrastructural alternations of tight junctions, including discontinuities and openings were identified in cerebral ischemia [56]. In this study, most of the tight junctions appeared intact as a series of electron-dense zones sealing intercellular clefts in sham control mice (Fig. 6a_1 − 2_). By contrast, ultrastructural abnormalities of tight junctions were observed in LIB-exposed mice, including discontinuities, membrane-bound spaces, and vacuole formations at 7 and 30 DPI (Fig. 6a_3 − 8_). Quantitative analyses revealed percentages of intact tight junctions were lower in LIB-exposed mice compared to sham controls at 7 DPI (52.72% vs. 80.00%) and 30 DPI (51.51% vs. 78.05%) (Fig. 6b).

### Levels of neurofilament light chain (Nf-L) and glial fibrillary acidic protein (GFAP) in mouse brain tissues and plasma post-LIB exposure

Brain tissue and circulating levels of neuronal and glial protein alterations in LIB-exposed mice were further estimated using the assays of FDA-approved biomarkers in TBI patients—Nf-L and GFAP. It was previously hypothesized that elevated serum levels of neuron- or glial-specific proteins, including neurofilaments indicate increased permeability of the BBB in blast-induced TBI, in addition to neuronal and glial cell damage or loss [57]. It was also reported that higher levels of serum neurofilament levels were associated with greater impairment of BBB integrities in multiple sclerosis[58]. Tissue Nf-L was significantly elevated by 36.3% (mean values) in LIB-exposed mice compared to sham controls at 1 DPI, but not at 7 DPI and 3 months post injury; while plasma Nf-L was significantly elevated by 658.8% (mean values) in LIB-exposed mice compared to sham controls at 3 months post injury, but not at 1 and 7 DPI (Supplemental Fig. 2). In contrast no significant differences of tissue or plasma GFAP were identified in LIB-exposed mice compared to sham controls at 1, 7 DPI and 3 months post injury (Supplemental Fig. 3).

## Discussion

Previous NVU studies of bTBI mainly focused on moderate-to high-intensity blast exposure, repetitive blast exposure, or in conjunction with polytrauma [17, 20, 21, 59–64]. Since the majority of brain injuries in military personnel are classified as mild TBI [65], evaluations of NVU consequences in response to a single LIB provide an understanding of the pathophysiological impacts of mild TBI to help design therapeutic targets to minimize these injuries. In our recently established open-field, non-inertial LIB mouse model with peak overpressure of 46.6 kPa, no brain damage was readily visible under light / fluorescence microscopes. However, nanoscale ultrastructural abnormalities, including defects in axonal myelin sheaths, mitochondria, and synapses were detected in LIB-exposed mice [23–25]. The present study clearly demonstrates damage in multiple cellular components of the NVU at the ultrastructural level, including luminal circularities of microvessels, pericytes, endothelial cells, basement membranes, astrocyte end-feet, and tight junctions post-LIB exposure, as well as vasomotor patterns with quantitative proteomics.

The UCLA Brain Injury Research Center Program reported that 45.2% of TBI patients had vasospasm from 2 to 13 DPI [66]. The incidence of vasospasm is also very high in patients with bTBI. Operation Iraqi Freedom (OIF) reported 47.4% of TBI patients (majority bTBI) suffered from vasospasm up to 30 DPI and with an average spasm duration of 14.3 DPI. Continued vasospasm was associated with higher mortality [67]. Using an in vitro blast model, Patrick W. Alford et al. demonstrated vascular hypercontractility and vascular smooth muscle phenotype switching [68]. Vasospasm was also identified in animal models of high-intensity blast exposure [21, 69, 70]. Here we extended studies to include details of luminal circularities of microvessels at the ultrastructural level. Quantitative analyses of electron micrographs revealed decreased lumen areas and increased lumen ratios in LIB-exposed mice at 7 DPI, indicating microvessel constrictions that correlate with these preclinical / clinical observations (Fig. 1).

The contraction of pericytes as the key regulator of microvessel diameters is Ca^2+^ dependent; thus, a rise of Ca^2+^ may evoke the constrictions of microvessels [71, 72]. Our proteomic investigation revealed significant upregulation of calcium-transporting ATPase (two isoforms) in LIB-exposed mice at 24 h post injury (Supplemental Fig. 1). It is reasonable to hypothesize that the disruption of Ca^2+^ hemostasis may be one of the mechanisms responsible for acute microvessel constrictions post-LIB exposure. Pericyte degeneration and disrupted pericyte-endothelial cell integrity also occur in TBI [73, 74]. Disruption of Ca^2+^ hemostasis may promote pericyte degeneration in LIB-exposed mice (Fig. 2). Compromised contractility of pericytes may thus contribute to microvessel dilation in LIB-exposed mice at 30 DPI (Fig. 1). Time-dependent cerebral vascular hyperreactivity in response to vasoactive mediators is another possible mechanism underlying vasomotor dysregulation [70]. Our proteomic study revealed significant downregulation of tyrosine 3-monooxygenase, serine / threonine-protein phosphatase (fragment) and serine / threonine-protein phosphatase PP1-alpha catalytic subunit, especially the predominant reduction of nitric oxide synthase (NOS) by 43.3% in LIB-exposed mice (Supplemental Fig. 1). These changes are consistent with previous reports, suggesting the NOS pathways play important roles in modifying cerebral blood flow after TBI [17, 75–78]. Further studies targeting Ca^2+^ and / or the NOS pathways may offer promising therapeutic strategies to mitigate the microvascular effects of LIB-induced mTBI.

Diffuse cerebral edema is one of the characteristic features of bTBI [79–82]. Herein, we identified swellings of multiple NVU components induced by single LIB exposure (Figs. 3, 4 and 5). AQP-4, one of the most abundant water channels in the brain, is highly polarized to perivascular astrocytic end-feet. Altered expression and / or polarization of AQP-4 have been linked, both in experimental models and in humans, to impaired water homeostasis in neurological diseases such as ischemic stroke and neurotrauma [83–85], including bTBI [22, 64, 86]. In a previous study, peaks of AQP-4 expression were identified at 12 and 72 h and then decreased at 15 days post TBI [87], which may explain the swellings of endothelial cells and astrocyte end-feet were relatively more severe at 7 days post-injury than that at 30 days post-injury in LIB-exposed mice. Reactive astrocytes have been shown to induce loss of AQP-4 polarization [88, 89]. Herein, perivascular astrocytes showed somal hypertrophy and increased expression of GFAP –characteristic features of reactive astrocytes in LIB-exposed mice (Fig. 5) [54]. Reactive astrogliosis-associated AQP-4 modulation can be considered a potential mechanism of LIB-induced edema of NVU components. A previous immunogold study demonstrated that pericytes regulate AQP-4 anchoring to perivascular astrocyte end-feet [90]. Reduction of a pericyte marker, platelet-derived growth factor receptor β, has been linked to a loss of AQP-4 polarization and suppressed glymphatic system [91]. Our finding of pericyte degeneration in LIB-exposed mice (Fig. 2) suggests a potential role of dysregulated pericyte-mediated AQP-4 polarization as another possible mechanism underlying LIB-induced edema of NVU components.

The structural integrity and interactions between different NVU components are essential to the regulations of microvascular permeability and the dynamics of cerebral microcirculation [11, 92]. Tight junctions play essential roles in maintaining the integrity of the NVU by connecting adjacent endothelial cells. Reduced expressions of tight junction-specific proteins, including ZO-1, claudin-5, and occludin, have been reported in bTBI [64, 93–96]. In our current study, high magnification images captured by TEM helped us to confirm that single LIB exposure was able to induce ultrastructural abnormalities of tight junctions, such as tight junction discontinuities, membrane-bound spaces, and within vacuole formations (Fig. 6). Moreover, we found significantly enlarged distances between astrocyte end-feet and basement membranes (Fig. 5), indicating possible detachment of the NVU in the LIB-exposed mice.

The impaired integrity of NVU resulting in BBB damage in LIB-exposed mice was further supported by elevated circulating levels of Nf-L at 3 months post LIB exposure (Supplemental Fig. 2). It was demonstrated that BBB integrity was inversely proportional to the concentration of Nf-L in the plasma of humans [58]. Circulating levels of Nf-L are well-documented in clinical studies as potential biomarkers of BBB damage and may predict clinical outcomes in TBI patients, including in the military population [97, 98]. Further studies estimating serum Nf-L alterations with therapeutic interventions in LIB models may aid in bench-to-bedside translational research of bTBI. In addition, we identified significantly elevated levels of Nf-L in acute phase (24 h) post LIB exposure (Supplemental Fig. 2). We speculated that NF-L might have been subjected to partial proteolysis[99, 100] and released into the more extractable pool. Though there were no significant changes in levels of the astrocytic biomarker GFAP in both cortical tissues and plasmas compared to sham controls (Supplemental Fig. 3) and by quantifying the fluorescent intensities of GFAP in the brain Sect. [24], as expected for the primary LIB conditions resulting in such “*invisible injury*”, we indeed observed structural abnormalities of tight junctions and astrocyte end-foot detachment from basement membranes noted in the LIB-exposed mice (Fig. 5), supporting the notion of impaired NVU integrity post LIB exposure.

The extracellular matrix (ECM) is the biological scaffold material that maintains the integrity of the NVU by providing support and interactions between different NVU components [92, 101]. Activated matrix metalloproteinases (MMPs), especially gelatinases (MMP-2 / 9) were reported to mediate the degradation of ECM and tight junctions, leading to impaired integrity of the NVU [11, 102–105]. Our previous publications have demonstrated that SB-3CT, a specific gelatinase inhibitor, was able to restore NVU integrity, and subsequently, neurological functions in mice with severe TBI and ischemic stroke [11, 53, 102, 103]. Considering that upregulation and activation of gelatinases have been reported in bTBI by multiple research groups [22, 64, 106, 107], specific inhibition of gelatinases offers a potential therapeutic strategy in managing LIB-induced NVU damage.

## Conclusion

Overall, our current study provides comprehensive quantitative and qualitative assessments of LIB-induced multi-component NVU abnormalities, potentially leading to widespread BBB impairments. The open-field blast model offers a potential platform for testing vascular interventions to minimize or mitigate primary LIB injury.

### Electronic supplementary material

Below is the link to the electronic supplementary material.

**Supplemental Fig. 1.** Proteomic vasomotor alterations in mouse brains post-LIB exposure. Quantitative proteomics values (spectral counts) of vasomotor-related proteins with significant differences after LIB exposure. *, p < 0.05 and **, p < 0.01. Data are expressed as mean ± SD.

**Supplemental Fig. 2.** Tissue and plasma levels of neurofilament light (Nf-L) in mouse brains post-LIB exposure. Quantification of Nf-L in brain tissue and plasma of sham controls and LIB-exposed mice at 1, 7 days and 3 months post injury. Nf-L levels in tissues (pg/µg) and in plasma (ng/ml) at 1 and 7 DPI and 3 months post-LIB are expressed mean ± SD; other data are expressed as median and interquartile range. *, p < 0.05 and **, p < 0.01. 24-hour tissue sham: n = 4, LIB: n = 6; 24-hour plasma sham: n = 5, LIB: n = 5; 7-day tissue sham: n = 10, LIB: n = 4; 7-day plasma sham: n = 3, LIB: n = 4; 3-month tissue sham: n = 6, LIB: n = 6; 3-month plasma sham: n = 5, LIB: n = 6.

**Supplemental Fig. 3.** Tissue and plasma levels of glial fibrillary acidic protein (GFAP) in mouse brains post-LIB exposure. Quantification of GFAP in brain tissue and plasma of sham controls and LIB-exposed mice at 1, 7 days and 3 months post injury. Data are expressed as mean ± SD. 24-hour tissue sham: n = 4, LIB: n = 6; 24-hour plasma sham n = 5, LIB: n = 6; 7-day tissue sham: n = 10, LIB: n = 4; 7-day plasma sham n = 3, LIB: n = 2; 3-month tissue sham: n = 6, LIB: n = 6; 3-month plasma sham: n = 6, LIB: n = 6.

## Data Availability

The datasets used and/or analyzed during the current study are available from the corresponding author on request.

## Abbreviations

mTBI: mild traumatic brain injury
LIB: low-intensity blast
TEM: transmission electron microscopy
NVU: neurovascular unit
Nf-L: neurofilament light chain
GFAP: glial fibrillary acidic protein
DPI: days post-injury
bTBI: blast-induced traumatic brain injury
CDC: Centers for Disease Control and Prevention
TBICoE: Traumatic Brain Injury Center of Excellence
DoD: US Defense Department
BBB: blood–brain barrier
MMP: matrix metalloproteinases
ARRIVE: animal research:reporting of in vivo experiments
i.p. injection: intraperitoneal injection
AQP-4: aquaporin-4
GFAP: glial fibrillary acidic protein
SDS: sodium dodecyl sulfate
TEAB: tetraethylammonium bicarbonate
PASEF: parallel accumulation–serial fragmentation
MS: mass spectrometry
NOS: nitric oxide synthase
OIF: Operation Iraqi Freedom
ECM: extracellular matrix
