# Supplementary figures and images for: Low-intensity open-field blast exposure effects on neurovascular unit ultrastructure in mice
Source: Acta Neuropathol Commun. 2023 Sep 6;11:144. doi: 10.1186/s40478-023-01636-4 (PMC10481586; doi:10.1186/s40478-023-01636-4)

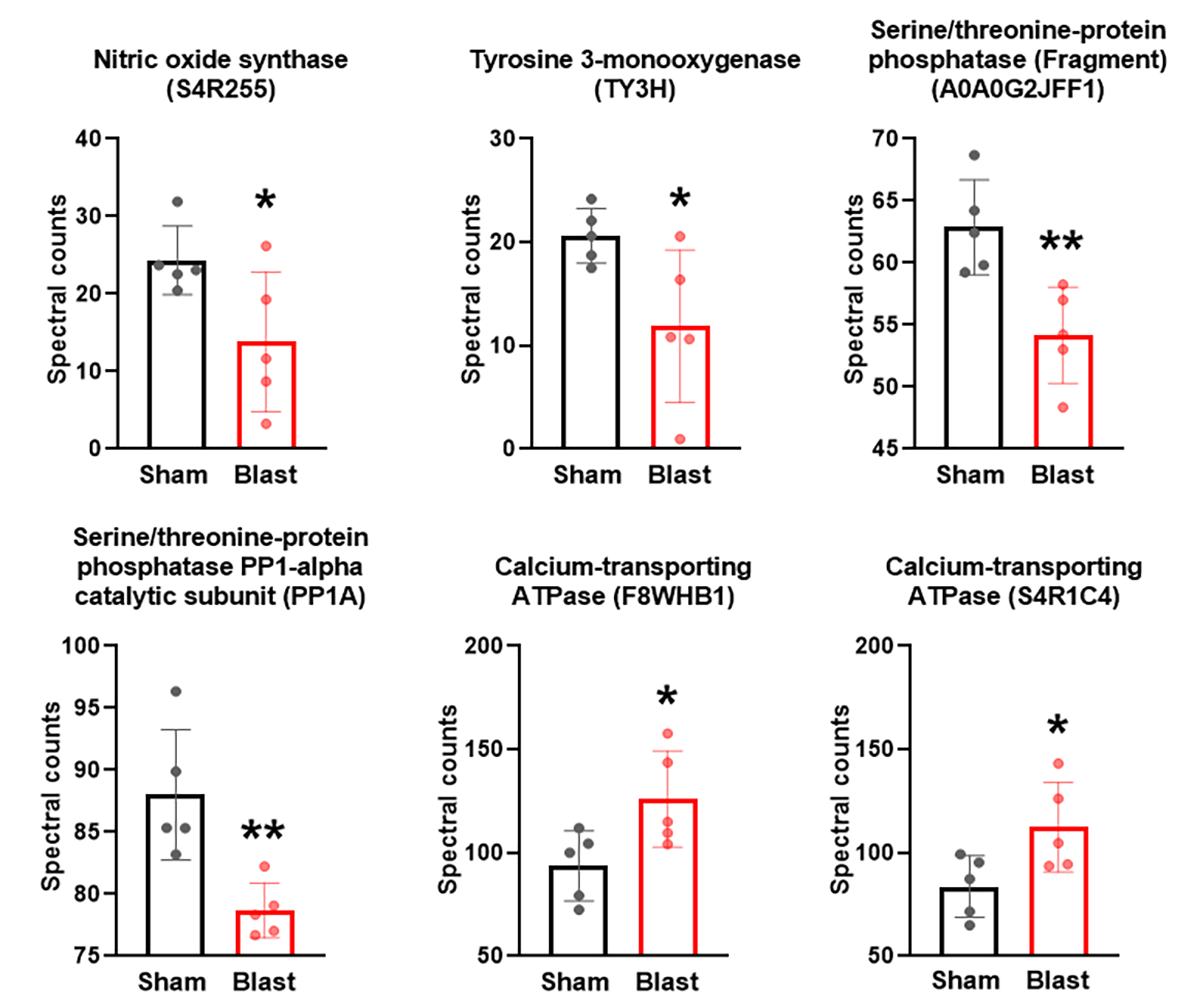

Supplement: Supplementary file 1 — Supplemental Fig. 1. Proteomic vasomotor alterations in mouse brains post-LIB exposure. Quantitative proteomics values (spectral counts) of vasomotor-related proteins with significant differences after LIB exposure. *, p < 0.05 and **, p < 0.01. Data are expressed as mean ± SD. [file 40478_2023_1636_MOESM1_ESM.png]

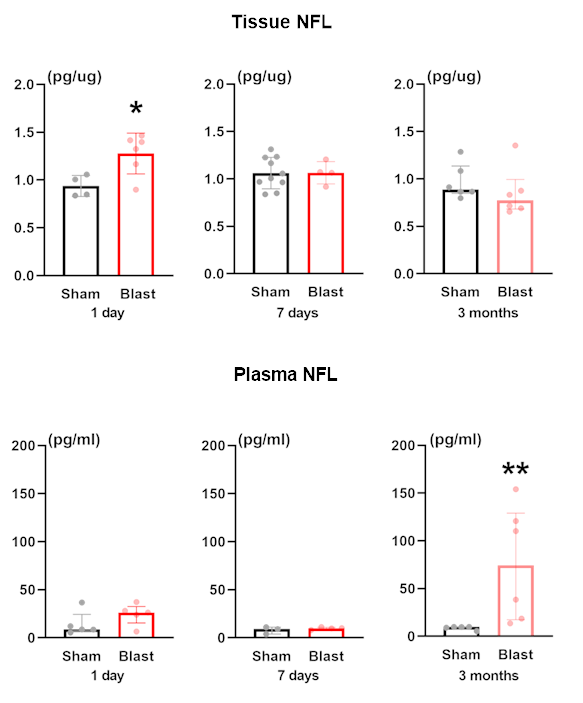

Supplement: Supplementary file 2 — Supplemental Fig. 2. Tissue and plasma levels of neurofilament light (Nf-L) in mouse brains post-LIB exposure. Quantification of Nf-L in brain tissue and plasma of sham controls and LIB-exposed mice at 1, 7 days and 3 months post injury. Nf-L levels in tissues (pg/µg) and in plasma (ng/ml) at 1 and 7 DPI and 3 months post-LIB are expressed mean ± SD; other data are expressed as median and interquartile range. *, p < 0.05 and **, p < 0.01. 24-hour tissue sham: n = 4, LIB: n = 6; 24-hour plasma sham: n = 5, LIB: n = 5; 7-day tissue sham: n = 10, LIB: n = 4; 7-day plasma sham: n = 3, LIB: n = 4; 3-month tissue sham: n = 6, LIB: n = 6; 3-month plasma sham: n = 5, LIB: n = 6. [file 40478_2023_1636_MOESM2_ESM.png]

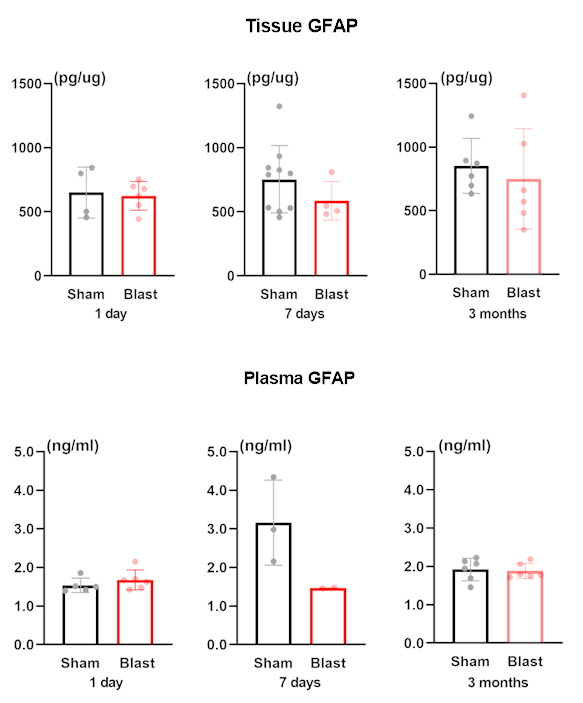

Supplement: Supplementary file 3 — Supplemental Fig. 3. Tissue and plasma levels of glial fibrillary acidic protein (GFAP) in mouse brains post-LIB exposure. Quantification of GFAP in brain tissue and plasma of sham controls and LIB-exposed mice at 1, 7 days and 3 months post injury. Data are expressed as mean ± SD. 24-hour tissue sham: n = 4, LIB: n = 6; 24-hour plasma sham n = 5, LIB: n = 6; 7-day tissue sham: n = 10, LIB: n = 4; 7-day plasma sham n = 3, LIB: n = 2; 3-month tissue sham: n = 6, LIB: n = 6; 3-month plasma sham: n = 6, LIB: n = 6. [file 40478_2023_1636_MOESM3_ESM.png]
